# IgG4-related kidney disease mimicking renal cell carcinoma: a case report and literature review

**DOI:** 10.3389/fmed.2026.1933790

**Published:** 2026-09-21

**Authors:** Huangyu Luo, Guang Han, Zhong Tian, Cheng Zhu, Zhouhui Chen, Qiang Xu, Xiaobin Jian, Yelian Cai, Qingqing Zhang, Bo Yu, Ni Fu

**Affiliations:** 1 Department of Urology, the Second Affiliated Hospital of Zunyi Medical University, Zunyi, China; 2 Department of Urology, the First Affiliated Hospital of Xinjiang Medical University, Urumqi, China; 3 Department of Pathology, the Second Affiliated Hospital of Zunyi Medical University, Zunyi, China

**Keywords:** CT, IgG4-RD, IgG4-RKD, RCC, renal mass

## Abstract

IgG4-related kidney disease (IgG4-RKD) presenting as renal masses is a very rare clinical entity. IgG4-RKD represents the renal manifestation of IgG4–related disease (IgG4-RD) and is characterized primarily by tubulointerstitial nephritis (TIN) caused by infiltration of IgG4-positive plasma cells in the renal interstitium and tubules. Typical imaging findings include renal enlargement and cortical low-density lesions; however, a very small proportion of IgG4-RKD cases may present as space-occupying renal lesions, which are easily misdiagnosed as renal malignancies. In this report, we describe a patient hospitalized for a respiratory disease whose abdominal computed tomography (CT) scan (non-contrast, contrast-enhanced, and 3D reconstruction) revealed a space-occupying lesion in the right kidney, with no significant abnormalities in the left kidney. Clinicians initially suspected a malignant renal tumor and proceeded with radical nephrectomy of the right kidney. Postoperative histopathology and immunohistochemistry confirmed the diagnosis of IgG4-RKD.

## Introduction

IgG4-RD is a group of fibroinflammatory disorders that can affect multiple organs (1), a concept first proposed in 2003. It is now well established that this condition can involve a wide range of organs, including the pancreas (autoimmune pancreatitis), salivary glands (sialadenitis), kidneys (TIN),lacrimal apparatus (dacryoadenitis/dacryocystitis) (2). IgG4-RD primarily manifests as fibrosing inflammatory infiltration in the affected organs; in addition, it may lead to soft tissue hyperplasia within or around the involved organs, forming space-occupying lesions that mimic malignant tumors (3). The kidneys are among the more commonly affected organs.

IgG4-RKD was first described in 2004 as a general term for renal lesions associated with IgG4-RD. It is characterized by a distinctive pattern of TIN with dense plasma cell–rich infiltrates, typically accompanied by abundant IgG4-positive plasma cells and varying degrees of fibrosis (i.e., IgG4-related TIN or IgG4-TIN) (4, 5). Typical imaging findings include bilateral multiple low-attenuation or low-signal cortical lesions; among those who underwent contrast-enhanced CT, only approximately 3.4% of cases presented as solitary renal space-occupying lesions, which are clinically rare and easily misdiagnosed (6).

In this report, we describe a clinical case exemplifying this uncommon presentation. The patient was initially admitted to the respiratory department with pulmonary tuberculosis. During hospitalization, abdominal CT revealed a space-occupying lesion in the right kidney, and subsequent magnetic resonance imaging (MRI) suggested right renal cell carcinoma (RCC), leading to a preliminary diagnosis of right renal cancer. A laparoscopic radical nephrectomy of the right kidney was performed. Postoperative histopathological and immunohistochemical examinations confirmed IgG4-RKD. The patient declined further treatment with glucocorticoids and immunosuppressants and was discharged on postoperative day 9.

## Case report

A 60-year-old man developed a cough two months prior that did not respond to self-administered oral antibiotics. He was admitted to the Department of Respiratory Medicine at our hospital and diagnosed with secondary pulmonary tuberculosis. On admission, he reported no history of surgery, palpitations, or dyspnea.

During treatment, the patient underwent noncontrast and contrast-enhanced abdominal CT, which revealed a space-occupying lesion measuring approximately 43 × 33 × 45 mm in the midportion of the right kidney. The lesion had ill-defined margins and showed progressive enhancement after contrast administration, findings highly suggestive of RCC (Figure 1). The left kidney was normal in size, morphology, and density. No definite abnormalities were observed in other solid organs, including the pancreas, biliary tract, and liver. The salivary and lacrimal glands were not specifically examined because they were not part of the routine imaging protocol, and no clinical signs suggestive of IgG4-related sialadenitis or dacryoadenitis were present. Laboratory testing revealed a mildly elevated aspartate aminotransferase level (41 U/L) and a decreased prealbumin level (95 mg/L). All other hematologic parameters, renal function indices, and urinalysis results were within normal limits. Serum IgG4 levels were not measured preoperatively.

**Figure 1:**
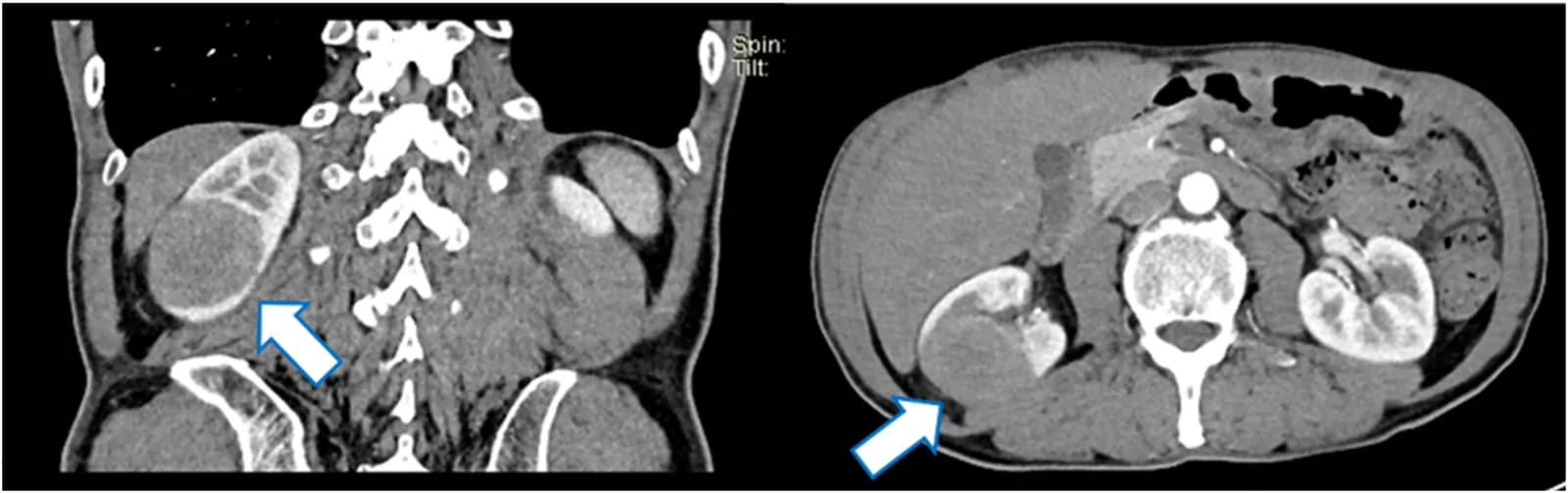
Abdominal CT shows no significant abnormalities in the left kidney, while a space-occupying lesion measuring approximately 43 × 33 × 45 mm is present in the right kidney.

After 1 month of antituberculous therapy, the patient's respiratory symptoms improved, and he was transferred to the Department of Urology for further evaluation. Noncontrast and contrast-enhanced MRI of the upper abdomen demonstrated a space-occupying lesion in the right kidney. The lesion measured approximately 33 × 48 mm, was isointense on T1-weighted images(T1WI), exhibited heterogeneous signal intensity on T2-weighted images(T2WI), and showed progressive enhancement after contrast administration. No significant abnormalities in morphology or signal intensity were observed in the left kidney, and no abnormalities were identified in other organs, including the pancreas and liver. Based on the CT and MRI findings, a right renal malignancy was strongly suspected (Figure 2), and the preliminary diagnosis was right-sided RCC.

**Figure 2:**
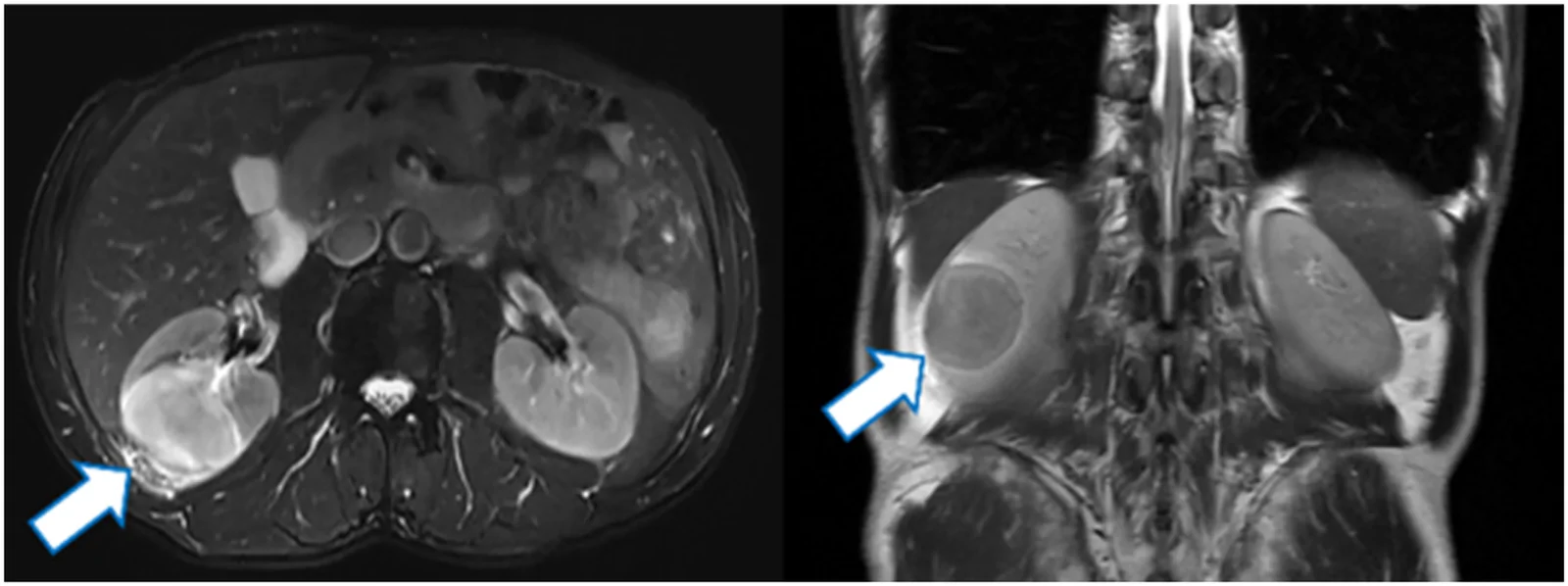
Upper abdominal MRI showed no obvious abnormalities in the left kidney. A space-occupying lesion was identified in the right kidney, exhibiting isointensity on T1WI and heterogeneous signal intensity on T2WI, raising suspicion of a malignant renal tumor.

Because the imaging findings were highly suggestive of RCC and IgG4-RKD was not included in the initial differential diagnosis, the patient underwent laparoscopic right radical nephrectomy following multidisciplinary evaluation and provision of informed consent. Macroscopic examination of the resected right kidney revealed a space-occupying lesion measuring approximately 4.0 × 5.0 cm in the midportion of the kidney, with a pale cut surface (Figure 3). Histopathologic examination revealed focal nodular proliferation of fibrous tissue within the renal parenchyma. Glomerular and tubular structures were absent within the nodules, and the interstitium showed extensive plasma cell infiltration with sparse lymphocytic infiltration (Figure 4). Immunohistochemical staining demonstrated extensive infiltration by IgG4-positive plasma cells, with >10 IgG4-positive plasma cells per high-power field (HPF) and an IgG4-positive/IgG-positive plasma cell ratio of >40% (Figure 5).

**Figure 3:**
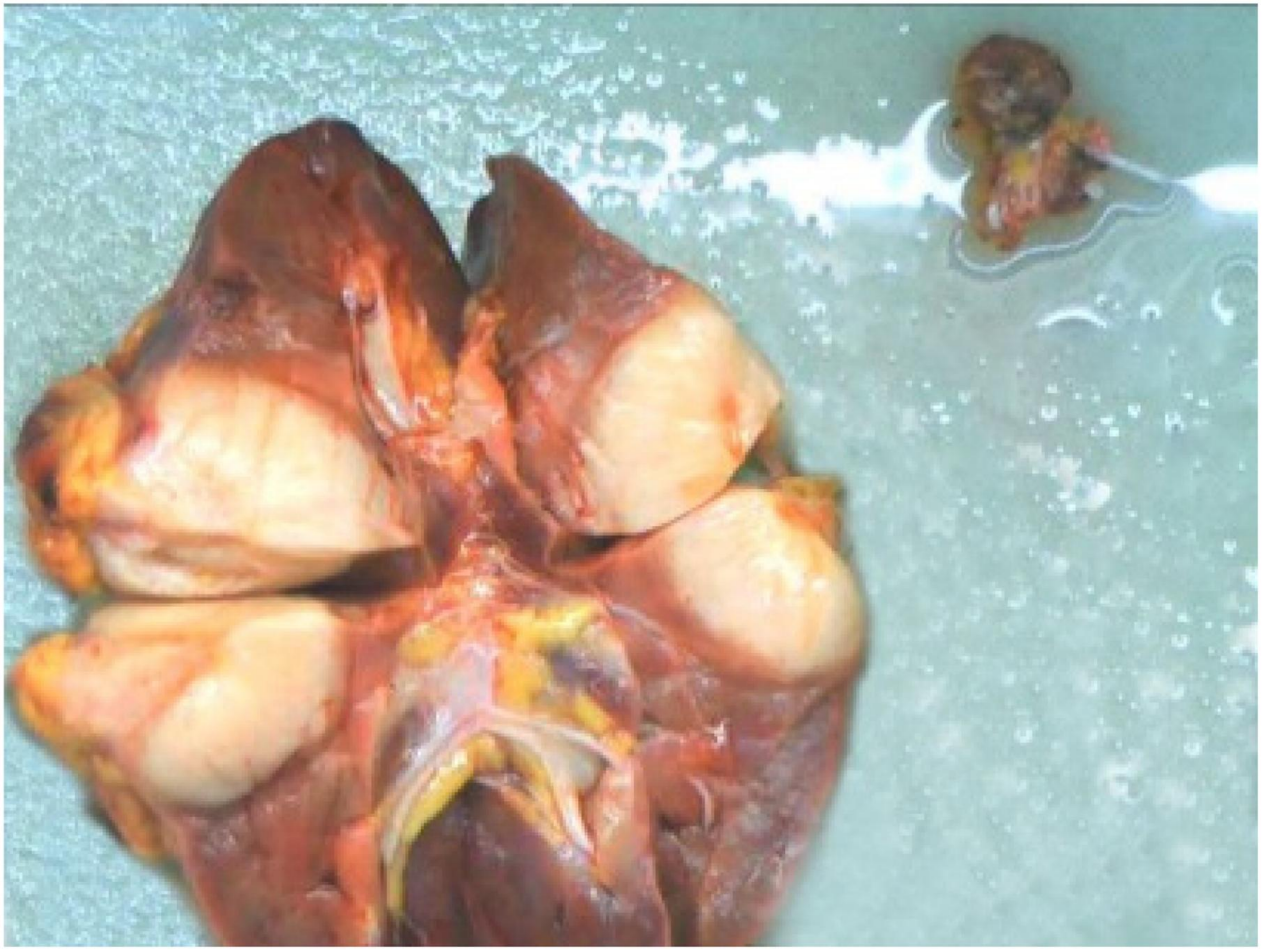
Intraoperative right kidney specimen showing a space-occupying lesion in the right kidney with indistinct boundaries from the surrounding renal tissue.

**Figure 4:**
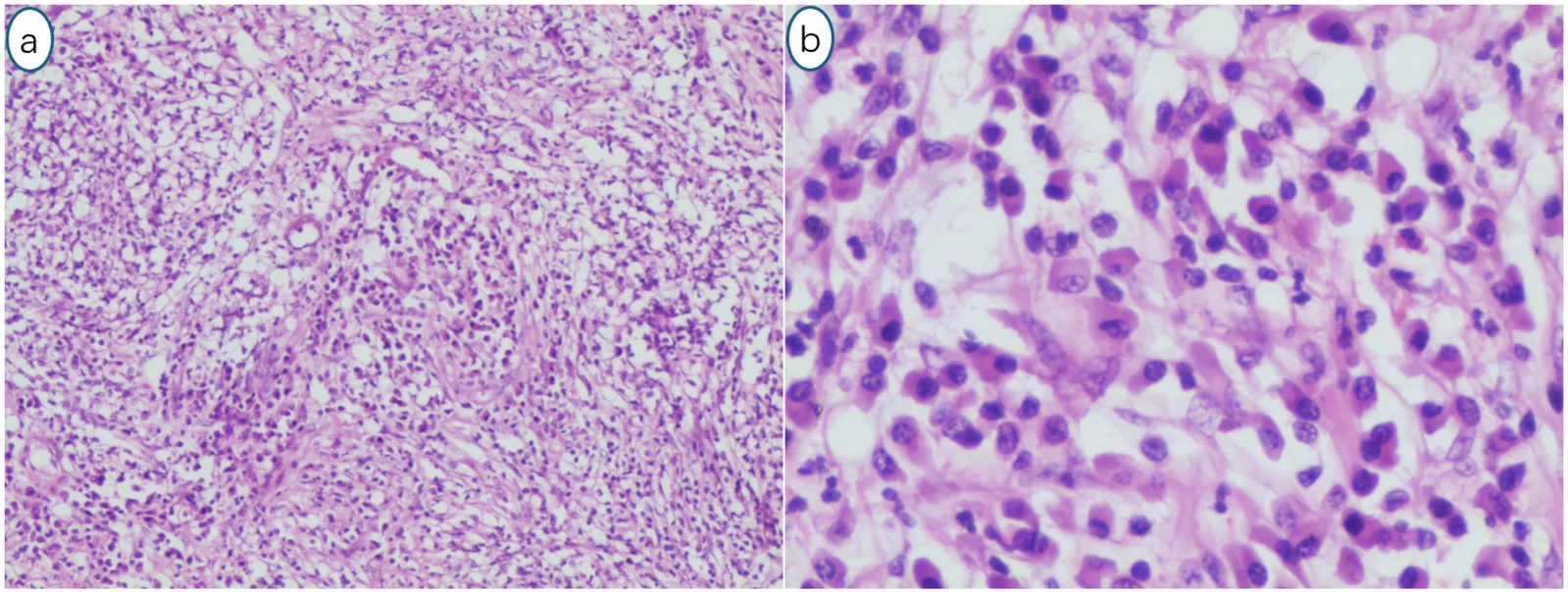
Focal nodular fibrous tissue hyperplasia is observed within the renal parenchyma; glomerular and tubular structures are absent within the nodules, with extensive plasma cell infiltration and minimal lymphocyte infiltration in the interstitium (**a**: HE   ×  10; **b**: HE   ×  40).

**Figure 5:**
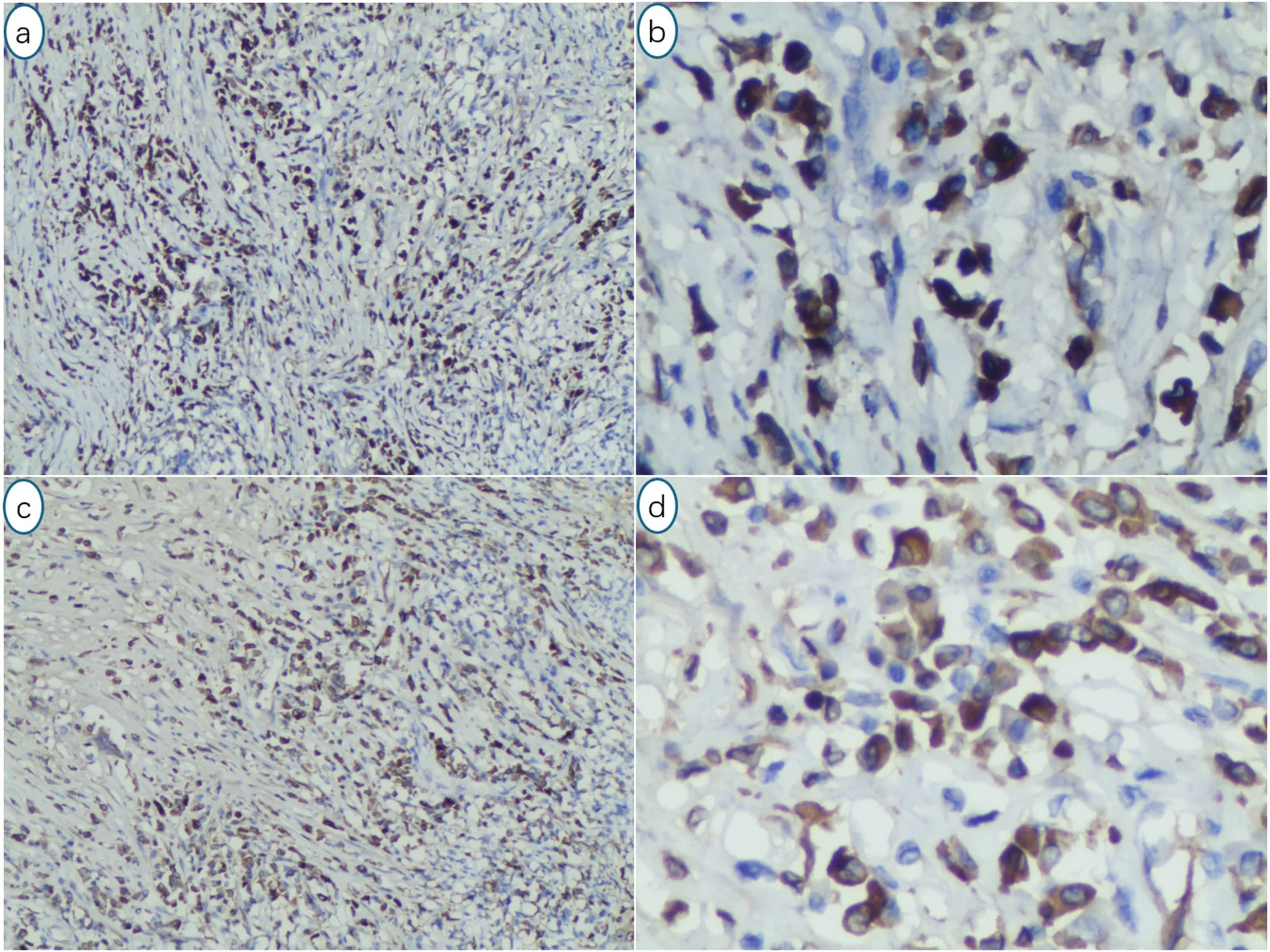
Extensive infiltration of IgG4-positive plasma cells is observed, with >10 IgG4-positive cells per high-power field (HPF) and an IgG4-positive/IgG-positive cell ratio exceeding 40% (**a**: IgG   ×  10; **b**: IgG   ×  40; **c**: IgG4 × 10; **d**: IgG4 × 40).

The patient's condition remained stable postoperatively. Based on the histopathologic and immunohistochemical findings, a diagnosis of IgG4-RKD was confirmed. The nephrology and rheumatology-immunology teams were consulted regarding intensive immunosuppressive therapy. Because the patient was also receiving treatment for pulmonary tuberculosis, an individualized assessment of the potential therapeutic benefits and infection-related risks was required. The patient declined additional treatment and elected to undergo close follow-up. He was discharged on postoperative day 9. As of the manuscript revision date, 13 months after surgery, the patient remained alive, and no definitive complications had been identified during follow-up. Although imaging may detect recurrence or new extrarenal involvement, incomplete longitudinal data on serum IgG4 and complement levels and renal function preclude conclusions regarding the natural course of the disease or the long-term efficacy of surgical resection.

## Discussion

IgG4-RD is a chronic, immune-mediated fibroinflammatory disorder characterized by tissue infiltration with IgG4-positive plasma cells. It can affect multiple organs and may result in progressive organ dysfunction (7, 8). In the urinary system, IgG4-RD may involve the renal parenchyma, tubulointerstitium, ureters, and bladder. TIN is the most common renal manifestation, although the disease may also be associated with retroperitoneal fibrosis (7). Most patients with IgG4-RD have involvement of one or more extrarenal organs. In the present case, however, the disease manifested solely as a solitary renal mass without evidence of extrarenal involvement, making the preoperative diagnosis particularly challenging.

Serologic testing in IgG4-RKD typically shows a marked elevation in serum IgG4 levels. Other laboratory abnormalities may include hypocomplementemia and mild renal impairment. However, some patients are asymptomatic and have no apparent laboratory abnormalities (9, 10). According to the 2011 diagnostic criteria for IgG4-RKD proposed by the Japanese Society of Nephrology (6), diagnosis is based on the following five components: (1) evidence of renal involvement, as indicated by abnormal urinalysis findings, impaired renal function, or other laboratory abnormalities; (2) characteristic renal imaging findings, such as multiple low-density lesions or a solitary renal mass; (3) an elevated serum IgG4 concentration (≥135 mg/dL); (4) renal histopathologic findings showing dense lymphoplasmacytic infiltration with fibrosis and >10 IgG4-positive plasma cells per HPF and/or an IgG4-positive/IgG-positive plasma cell ratio of >40%; and (5) histopathologic evidence of extrarenal organ involvement, characterized by >10 IgG4-positive plasma cells per HPF and/or an IgG4-positive/IgG-positive plasma cell ratio of >40%. Based on various combinations of these criteria, cases are classified as definite, probable, or possible IgG4-RKD. In 2021, a working group of the Japanese Society of Nephrology externally validated and revised these criteria. The updated criteria retain the original five-component framework while refining individual items and further emphasizing the central role of renal histopathologic evidence in establishing the diagnosis (11).

The imaging manifestations of IgG4-RKD are diverse and primarily include (1) bilateral, patchy, low-attenuation lesions in the renal cortex; (2) a solitary renal parenchymal mass; and (3) diffuse bilateral renal enlargement or renal capsular thickening. However, current imaging techniques often cannot reliably distinguish RCC from a solitary mass-forming lesion associated with IgG4-RKD (7). On contrast-enhanced CT, IgG4-RKD typically presents as one or more hypoenhancing renal lesions. On MRI, these lesions are generally isointense or hypointense on T1WI and hypointense on T2WI (12, 13).

To further characterize the clinical and imaging features of these lesions, we conducted a targeted literature review of the PubMed database from its inception (1946) to August 3, 2026, using combinations of the following keywords: “IgG4-related kidney disease”, “IgG4-related disease”, “renal mass”, “renal pseudotumor”, and “renal cell carcinoma”. The search was supplemented by manual screening of the reference lists of relevant publications. Studies were included if they (1) reported individual patients with histopathologically confirmed IgG4-RKD based on examination of renal tissue obtained by nephrectomy, partial nephrectomy, open biopsy, or percutaneous renal biopsy; (2) presented a unilateral renal mass as the dominant imaging finding; and (3) provided individual case-level data. Studies were excluded if they reported predominantly bilateral or multiple renal masses, non-renal (e.g., ureteral) masses, or reviews and case series without individual-level data. Twelve patients from 12 English- or Japanese-language studies published between 2007 and 2025 met these criteria (Table 1). The patients ranged in age from 53 to 80 years, with a mean age of 64.3 years. Six patients were male and six were female, yielding a male-to-female ratio of 1:1. All lesions presented as unilateral renal masses as the dominant imaging finding: nine were located within the renal parenchyma, two primarily involved the renal pelvis, and one was located at the renal hilum.

**Table 1:** Reported cases of IgG4-RKD presenting predominantly as unilateral renal masses (2007–2025).

No.	First author, year	Age, years/Sex	Renal involvement	Renal imaging findings	Serum IgG4 level	IHC-IgG4 + plasma cells/HPF	IHC-IgG4+/IgG + plasma-cell ratio	Renal Surgical Treatment	Medical treatment	Extrarenal involvement	Follow-up duration and findings	Ref
1	Cornell et al., 2007 (25)	57/M	Left; solitary	CT: left renal mass MRI: NR	NR	>10/HPF	NR	nephrectomy	NR	Pancreas Submandibular gland	NR	(25)
2	Shoji et al., 2010 (26)	56/F	Left; solitary	CT: left renal tumor, low-attenuation, delayed enhancement; MRI: left renal tumor, T1 isointense, T2 hypointense	Preoperative: NR Postoperative: Within reference range (68.9 mg/dL)	>10/HPF	NR	Radical nephrectomy	NP	None	Duration 12 months; Stable, no recurrence	(26)
3	Stamatakis et al., 2012 (27)	60/F	Right; solitary	CT: 3.4 × 3.0 cm, enhancing mass located in the posterior mid-pole cortex of the right kidney MRI: NR	NR	>10/HPF	NR	Partial nephrectomy	NR	NR	NR	(27)
4	Nofuji et al., 2013 (28)	56/M	Left; solitary	CT: 28 mm buried left renal hilar mass, early enhancement, delayed washout MRI: NR	Preoperative: NR Postoperative: Elevated (261 mg/dL)	NR	>40%	Partial nephrectomy	NP	Prostate	NR	(28)
5	Fukuhara et al., 2014 (29)	63/F	Right; solitary with contralateral lesions	CT: 31 × 24 mm cystic tumor at right upper pole; MRI: NR	Preoperative: Elevated (218 mg/dL) Postoperative: The serum IgG4 level normalized to 28.1 mg/dL after 6 months of corticosteroid therapy.	>10/HPF	>40%	Laparoscopic Right Nephrectomy	Prednisolone	Pancreas Lungs	Duration 6 months; Improved	(29)
6	Zhang et al., 2015 (30)	53/F	Right; solitary	CT: soft-tissue mass at right renal pelvis, irregular border, mild homogeneous enhancement MRI: NR	Preoperative: NR Postoperative: Decrease in 2 months(325→245 mg/dL)	NR (not quantified; described as numerous IgG4 + plasma cells)	>40%	Right ureteronephrectomy	NP	None	Duration 2 months; Stable, no recurrence	(30)
7	Cai et al., 2016 (31)	80/F	Left; solitary	CT: left renal mass; MRI: NR	Preoperative: NR Postoperative (2 weeks): Within reference range (69.2 mg/dL)	NR	>40%	Radical nephrectomy	NP	None	Duration 3 months; Stable, no recurrence	(31)
8	Park et al., 2016 (32)	75/M	Right; solitary (renal pelvis)	CT: mass at right renal pelvis; MRI: T1 isointense, T2 hypointense	Preoperative: NR Postoperative: Within reference range (78.6 mg/dL)	>10/HPF	>40%	Nephroureterectomy	NP	None	Duration 10 months; Stable, no recurrence	(32)
9	Bianchi et al., 2017 (33)	56/M	Right; solitary	CT: right renal mass(12 × 9 × 8 cm); MRI: right renal mass	Preoperative: NR Postoperative: Within reference range (69 mg/dL)	NR	>40%	Nephroureterectomy	Prednisone plus azathioprine	NR	Duration 18 months; Improved	(33)
10	Inoue et al., 2018 (34)	74/M	Right; pseudotumor and bilateral rim-like lesions	CT: right renal mass with bilateral rim-like lesions MRI: NR	Preoperative: Elevated (1,120 mg/dL) Postoperative: Decrease (25.6 mg/dL)	>10/HPF(232/HPF)	>40%	Open biopsy of the mass in the upper pole of the right kidney	Prednisolone	bile duct, pancreas, stomach	Duration 2 months; Improved	(34)
11	Watanabe et al., 2019 (35)	69/F	Right; solitary	CT: solitary renal mass; MRI: T1/T2 hypointense	Preoperative: Elevated (295 mg/L) Postoperative: NR	>10/HPF(600/HPF)	>40%	Partial nephrectomy	NR	IgG4-related Mikulicz disease (history)	NR	(35)
12	Minezaki et al., 2025 (23)	73/M	Left; solitary with diffuse enlargement	CT: Left diffuse renal enlargement with cystic mass; MRI: T2 hyperintense DWI heterogeneous	Elevated (425 mg/dL)	>10/HPF	15%	Percutaneous renal biopsy	Prednisolone	Stomach Pancreas Lymph node	Duration 12 months; Improved	(23)

M, male; F, female; R, right; L, left; B, bilateral; NR, not reported in the original article; ND, not determined or measured; NP, not performed; HPF, high-power field; T1WI, T1-weighted imaging; T2WI, T2-weighted imaging; ‘None’ indicates that the original article explicitly stated that no extrarenal involvement was identified;.

1. All patients included in this table had histopathologically confirmed IgG4-related kidney disease (IgG4-RKD) based on examination of renal tissue obtained by nephrectomy, partial nephrectomy, open biopsy, or percutaneous renal biopsy.

2. Serum IgG4 status (elevated or within the reference range) was determined according to the reference range reported in each original article or the cutoff value of 135 mg/dL specified in the diagnostic criteria proposed by Kawano et al. (6).

3. Immunohistochemical (IHC) findings are presented as reported in the original articles; qualitative descriptions (e.g., “numerous” or “abundant”) were not converted to numerical thresholds.

4. Cases are arranged chronologically by year of publication.

In the case reported by Watanabe et al., the patient had received continuous immunotherapy for preexisting Mikulicz disease for 10 years before the renal mass was diagnosed; however, the specific therapeutic agent was not reported. No additional medical therapy was documented after the diagnosis of IgG4-RKD. Therefore, medical treatment was recorded as not reported (NR).

The outcome category “**Improved**” required a documented pretreatment abnormality—such as flank or abdominal pain, elevated serum IgG4 levels, hypocomplementemia, active lesions on imaging, renal dysfunction, or abnormal urinary biomarkers—that objectively improved or resolved during follow-up. Improvement was defined as symptom relief, normalization or a marked reduction in serum IgG4 levels, regression or resolution of lesions, or improvement in renal function or urinary biomarkers.

The outcome category “**Stable, no recurrence**” was applied to patients with no documented pretreatment abnormality that could be assessed for improvement. These patients were asymptomatic, their renal masses were detected incidentally, and their serum IgG4 levels and other biomarkers were either within the reference ranges or not measured preoperatively. During follow-up, they experienced no recurrence, disease progression, or new extrarenal involvement and required no additional therapy. In the case reported by Zhang et al., serum IgG4 was first measured postoperatively and decreased from 325 to 245 mg/dL but remained above the reference range. Because no pretreatment baseline value was available, this case was classified as “**Stable, no recurrence**.”.

One patient (Watanabe et al.) was reported to have an elevated serum IgG4 level in the original article (295 mg/L), although this value corresponds to approximately 29.5 mg/dL and would be within the normal range by the 135 mg/dL cutoff.

On CT, most lesions were hypoattenuating and hypovascular relative to the normal renal parenchyma, with less pronounced enhancement than that of the normal renal cortex. Three lesions showed relatively marked enhancement, whereas two presented as cystic masses. Five patients underwent renal MRI, and signal characteristics were reported in four. Among the three patients with solid masses, the lesions were isointense on T1WI in two and hypointense in one, with the latter finding attributed to hemorrhage; all three lesions were hypointense on T2WI. In the remaining patient, the cystic lesion was hyperintense on T2WI. One additional patient reported by Bianchi et al. in 2017 underwent MRI, but the specific signal characteristics were not described.

Overall, the reported imaging findings were highly heterogeneous, precluding the identification of a consistent imaging profile. Although most lesions were described as hypoattenuating and hypovascular on CT, the limited MRI data included T2-hypointense solid lesions and a T2-hyperintense cystic lesion, and contrast enhancement was reported in only a few cases, as well as cystic lesions and atypical enhancement patterns. Although these findings have occasionally been cited as “typical” manifestations of IgG4-RKD, the extremely small sample size and inconsistent imaging protocols and reporting methods preclude estimation of their frequency or diagnostic performance and prevent their use as reliable discriminatory criteria.

RCC is among the 10 most common malignancies worldwide, with clear cell RCC(ccRCC) accounting for approximately 75% of cases (14). The classic clinical triad of RCC consists of flank pain, gross hematuria, and a palpable abdominal mass. However, with the widespread use of imaging, the proportion of patients presenting with this triad has decreased substantially, and most RCCs are now detected incidentally in asymptomatic patients (14). RCC subtypes exhibit distinct imaging characteristics. ccRCC typically appears as a hypervascular tumor with marked, often heterogeneous enhancement on contrast-enhanced CT and frequently demonstrates high signal intensity on T2WI. In contrast, papillary RCC (pRCC) is typically hypovascular, with mild or progressive enhancement on contrast-enhanced CT and low signal intensity on T2WI. Conversely, chromophobe RCC (chRCC) typically appears as a moderately vascular, well-circumscribed, and relatively homogeneous tumor, with moderate enhancement on contrast-enhanced CT/MRI and usually intermediate to low signal intensity on T2WI (15–17). The imaging features of RCC partially overlap with those of IgG4-RKD, potentially contributing to misdiagnosis in clinical practice. Although CT is valuable for the diagnosis and differential diagnosis of RCC, establishing a definitive diagnosis based on CT findings alone remains challenging. When clinically appropriate and imaging findings do not permit a definitive diagnosis, percutaneous renal biopsy should be considered (18).

In 2011, Kawano et al. reported a cohort of 41 patients diagnosed with IgG4-RKD between 2004 and 2011. Among those who underwent contrast-enhanced CT, only one patient (3.4%) presented with a solitary renal mass mimicking a malignant tumor (6), highlighting the rarity of this imaging phenotype. In the present case, both CT and MRI demonstrated a large space-occupying lesion in the right kidney that was highly suspicious for malignancy. Current guidelines from the American Urological Association (AUA) and European Association of Urology (EAU) recommend renal mass biopsy only when the results are expected to influence clinical management. If imaging findings strongly suggest malignancy and surgical resection is planned irrespective of the biopsy results, routine preoperative biopsy is generally not indicated (19, 20). Similarly, the EAU guidelines require pathological confirmation before ablative or systemic therapy when no prior pathological diagnosis is available but do not mandate biopsy before surgical resection of a suspected solid renal malignancy (21). Surgical resection remains the preferred curative treatment for localized RCC (20).

In this case, imaging revealed a solitary solid renal mass highly suggestive of RCC. Abdominal imaging showed no abnormalities of the pancreas, biliary tract, or retroperitoneum, and there were no clinical findings suggestive of an inflammatory or hematologic disorder. Therefore, in accordance with the aforementioned guidelines, proceeding directly to surgical resection was considered appropriate based on the information available at the time. Nevertheless, this case highlights the importance of considering IgG4-RKD in the differential diagnosis of a solitary renal mass. Failure to recognize this rare presentation narrowed the differential diagnosis and precluded further evaluation with serological testing and percutaneous renal biopsy, which might have enabled a preoperative diagnosis and potentially avoided unnecessary surgery.

Among the 12 cases reviewed, the renal lesions were predominantly incidentally detected as solitary or mass-like abnormalities, which may explain why they were frequently misdiagnosed as RCC. No patient presented with gross hematuria, and urinary symptoms attributable to the renal mass were absent in 10 of the 12 patients (83.3%). Approximately half had concomitant extrarenal manifestations of IgG4-RD, most commonly involving the pancreas. Such extrarenal involvement may provide important clues to the diagnosis during preoperative evaluation. In the present case, however, no definite extrarenal involvement was identified, further increasing the likelihood that IgG4-RKD presenting as a solitary renal mass would be overlooked in the preoperative differential diagnosis. Therefore, in patients with a renal mass, a thorough medical history should be obtained, with particular attention to previous or concurrent conditions such as autoimmune pancreatitis, IgG4-related sclerosing cholangitis, IgG4-related dacryoadenitis and sialadenitis, and retroperitoneal fibrosis. Careful assessment of the relevant extrarenal organs on imaging may further raise clinical suspicion for IgG4-RD and facilitate an accurate preoperative diagnosis.

Elevated serum IgG4 levels may support a diagnosis of IgG4-RD; however, this finding lacks specificity because IgG4-associated immune responses can also occur in other immune-mediated disorders, including pemphigus, membranous nephropathy, and thrombotic thrombocytopenic purpura. Therefore, the diagnosis of IgG4-RD should be based primarily on characteristic histopathological findings interpreted in conjunction with the clinical and imaging manifestations (22).

Of the 12 patients included in this study, serum IgG4 results were reported for 10. Elevated levels were observed in six patients, whereas the other four had normal serum IgG4 levels measured postoperatively. Because preoperative measurements were unavailable for these four patients, their preoperative serum IgG4 status could not be determined; therefore, it would be inappropriate to conclude that 40% of the patients had normal serum IgG4 levels before surgery. Accordingly, serum IgG4 testing is most useful for raising diagnostic suspicion and identifying patients who may benefit from further histopathological evaluation. A normal serum IgG4 level does not exclude IgG4-RKD, whereas an elevated level alone is insufficient to establish the diagnosis.

We also descriptively compared the principal clinical and imaging characteristics of these 12 cases with those of CcRCC, PRCC and chRCC (Table 2). However, imaging parameters were incompletely and inconsistently reported in the source literature: MRI data were available for only five cases, T2WI signal characteristics were described in only four, and the reported contrast-enhanced CT protocols and enhancement patterns varied substantially. Consequently, the imaging findings summarized in the “IgG4-RKD” column should be regarded as fragmentary observations derived from the available literature rather than definitive or representative imaging features of the disease. This limitation highlights an important gap in current knowledge: reliable imaging-based criteria for differentiating mass-forming IgG4-RKD, particularly isolated renal lesions, from renal malignancies have yet to be established.

**Table 2:** Descriptive comparison of clinical and imaging features between RCC and solitary-mass-type IgG4-RKD.

Item	Renal cell carcinoma (RCC)	Solitary-mass-type IgG4-RKD (12 cases)
Age/sex	Median age at diagnosis 65 years, male predominance (16)	Mostly middle-aged/elderly, both sexes (53–80 years in our series)
Symptoms	Classic triad: flank pain, gross haematuria, palpable abdominal mass; now mostly incidental findings (14)	Mostly asymptomatic, incidental discovery
Serum IgG4	Not part of routine RCC evaluation; no established role in RCC diagnosis (14, 17)	Using 135 mg/dL as the cutoff (6): 6 elevated (all >135 mg/dL); 4 normal (all first measured postoperatively)
Other laboratory findings	Typically unremarkable; no characteristic immunologic abnormalities. Paraneoplastic manifestations such as hypercalcemia, fever, and erythrocytosis may occur (14).	Hypergammaglobulinemia, elevated serum IgE, hypocomplementemia (low C3/C4), and elevated urinary *β*2-microglobulin may be present (6, 9, 10, 12). Mild renal impairment may occur. Autoantibodies (ANA, RF) may be positive but are nonspecific (9).
Extrarenal involvement	Common metastases to lung, liver, bone, etc. (14)	6 cases had documented extrarenal involvement, pancreas most common
CT findings	CcRCC (70%–80%): mostly hypervascular with washout enhancement; PRCC (10%–15%): mostly hypovascular with progressive enhancement; chRCC (4%–5%): intermediate, homogeneous, moderate enhancement (15–17)	Mostly hypoattenuating and hypovascular relative to the renal cortex, with less pronounced enhancement (delayed or progressive in some cases); 3 showed relatively marked enhancement and 2 presented as cystic masses; enhancement features were not described in 4 cases (contrast-enhanced CT was not feasible in 1).
MRI findings	CcRCC: mostly high–intermediate T2 signal, heterogeneous; PRCC: mostly low T2 signal, homogeneous; chRCC: mostly intermediate T2 signal, homogeneous (15, 16)	Of the 4 cases with MRI signal descriptions: isointense on T1WI in 2 and hypointense in 1 (due to hemorrhage); all 3 solid masses were hypointense on T2WI, while the 1 cystic lesion was hyperintense on T2WI.
Renal biopsy	Recommended when the results may influence management (e.g., masses with atypical or infiltrative features, patients considering active surveillance, or before ablative/systemic therapy) (19–21).	Should be considered when IgG4-RKD is suspected based on serological or imaging clues (6, 11, 27). Immunohistochemistry for IgG and IgG4 should be performed (6). Among the 12 cases, 1 was diagnosed by open biopsy and 1 by percutaneous biopsy, both avoiding nephrectomy.
Treatment	Localized: surgery (partial/radical nephrectomy), ablation, or active surveillance (14, 20); metastatic: targeted therapy plus immunotherapy, refractory to conventional chemotherapy (14);	Glucocorticoids are first-line therapy. (3, 24)

(1) The imaging data in the “Solitary-mass-type IgG4-RKD (12 cases)” column were derived from the original case reports summarized in Table 1. (2) The three cases with relatively marked enhancement on CT were Stamatakis et al., 2012 (27); Cai et al., 2016 (31); and Watanabe et al., 2019 (35). (3) The two cases presenting as cystic masses were Fukuhara et al., 2014 (29) and Minezaki et al., 2025 (23). (4) CT enhancement features were not described in four cases (Cornell et al., 2007 (25); Park et al., 2016 (32); Bianchi et al., 2017 (33); and Minezaki et al., 2025 (23)); in the case of Minezaki et al., contrast-enhanced CT was not feasible because of impaired renal function. (5) MRI was performed in five cases (Shoji et al., 2010 (26); Park et al., 2016 (32); Bianchi et al., 2017 (33); Watanabe et al., 2019 (35); and Minezaki et al., 2025 (23)); signal characteristics were reported in four, whereas Bianchi et al. did not describe the signal features.

When IgG4-RKD manifests as a renal mass, its nonspecific imaging features often make it difficult to distinguish from renal malignancy on imaging alone (22). A comprehensive assessment incorporating clinical, serological, and histopathological findings is therefore essential. In the case reported by Minezaki et al., a renal mass was initially detected on imaging; however, preoperative serological testing revealed a markedly elevated serum IgG4 level (425 mg/dL), and a subsequent renal biopsy demonstrated more than 10 IgG4-positive plasma cells per HPF. These findings, together with the overall clinical context, led to a diagnosis of IgG4-RKD. The patient responded favorably to glucocorticoid therapy during follow-up, thereby avoiding potentially unnecessary surgery (23).

This case suggests that preoperative renal biopsy may influence management decisions in patients with a renal mass accompanied by markedly elevated serum IgG4 levels, hypocomplementemia, or other organ manifestations suggestive of IgG4-RD. Nevertheless, the available evidence is derived primarily from isolated case reports. It therefore does not support routine biopsy of all solitary renal masses, nor does it imply that biopsy will invariably obviate the need for surgical intervention.

Regarding treatment, IgG4-RD generally responds well to therapy, and corticosteroids are commonly used and effective (3, 24). Among the 12 cases reported in the literature, four patients (33.3%) received pharmacologic treatment after surgery or biopsy: three received prednisolone monotherapy, and one received prednisone in combination with azathioprine. Of the four patients who received glucocorticoid therapy, serum IgG4 levels declined significantly and corresponding radiographic lesions regressed or improved in the three patients with pretreatment elevations. Among the eight patients with available follow-up data, all had favorable outcomes. Notably, the patient in the present case declined both glucocorticoid therapy and other immunosuppressive treatments after surgery. No definitive radiographic evidence of recurrence or new extrarenal involvement was observed during the 13-month follow-up period. These findings indicate only that the patient's condition remained relatively stable during the limited follow-up period and are insufficient to establish the long-term efficacy of surgery for IgG4-RD presenting as a solitary renal mass. For patients who do not receive immunosuppressive therapy, regular monitoring of renal function, urinary parameters, serum IgG4 levels, and complement levels remains recommended, together with imaging surveillance of the contralateral kidney, the surgical site, and organs commonly affected by IgG4-RD.

Based on this case and our literature review, we propose the following clinical evaluation pathway. When an isolated renal mass is detected, IgG4-RKD should be considered in the differential diagnosis. The initial evaluation should include a comprehensive medical history and physical examination to identify findings suggestive of IgG4-RD, such as autoimmune pancreatitis, IgG4-related sclerosing cholangitis, dacryoadenitis, sialadenitis, or retroperitoneal fibrosis. Concurrently, patients should be evaluated for features suggestive of ccRCC, including gross hematuria, paraneoplastic syndromes, and venous tumor thrombus. Multiphase contrast-enhanced CT and/or multiparametric MRI should then be performed to assess the vascularity, enhancement pattern, signal characteristics, margins, and local invasiveness of the mass. The pancreas, bile ducts, retroperitoneum, and other sites commonly affected by IgG4-RD should also be evaluated. In patients with imaging findings characteristic of CcRCC, particularly venous tumor thrombus or evidence of metastasis, and no findings suggestive of IgG4-RD, management should proceed according to established diagnostic and therapeutic pathways for RCC. Oncologic treatment should not be delayed to pursue IgG4-RD screening that is not supported by current evidence.

When a renal mass demonstrates hypovascularity, delayed or progressive enhancement, low signal intensity on T2WI, indistinct margins, a thick capsule or peripheral rim, or a renal pelvic or hilar location, additional testing may be warranted even in the absence of extrarenal manifestations of IgG4-RD. Such testing may include serum IgG4, total IgG and IgG subclass, IgE, and complement C3 and C4 levels; renal function tests; urinary biomarkers; and urinary *β*2-microglobulin measurement. Autoantibodies, such as antinuclear antibodies (ANA) and rheumatoid factor (RF), may aid in the differential diagnosis of other immune-mediated diseases but are not specific diagnostic markers for IgG4-RD. If the clinical, imaging, and serologic findings remain inconclusive and the histopathologic findings could alter the treatment strategy, percutaneous renal mass biopsy may be considered when technically feasible. Immunohistochemical staining for IgG and IgG4 should be performed, and the number of IgG4-positive plasma cells and the ratio of IgG4-positive to IgG-positive plasma cells should be documented. If the initial biopsy is nondiagnostic, a multidisciplinary team comprising urologists, nephrologists, radiologists, and pathologists should assess the oncologic risk, specimen adequacy, and patient preferences to determine whether repeat biopsy, close surveillance, or surgical intervention is appropriate. The diagnosis should not be based solely on individual imaging findings.

This report has several limitations. First, as a single-case report, its findings have inherently limited generalizability. Second, after postoperative consultation with the nephrology and rheumatology departments, intensified immunosuppressive therapy was considered. However, because the patient was concurrently receiving treatment for pulmonary tuberculosis, the use of glucocorticoids and other immunosuppressive agents required an individualized assessment of the potential therapeutic benefits versus the risks of inadequate infection control, tuberculosis activation or recurrence, and other infection-related complications. After the proposed treatment and its potential benefits and risks had been fully discussed, the patient declined further intensified immunosuppressive treatment and chose close follow-up. Nevertheless, untreated IgG4-related disease may still carry long-term risks, including disease recurrence or progression, involvement of other organs, and deterioration of renal function. Therefore, disease activity, relevant serological parameters, renal function, and potential involvement of other organs should be continuously monitored during follow-up, and the appropriateness of immunosuppressive therapy should be reassessed according to the patient's clinical course. Third, the follow-up period was limited to 13 months, which was insufficient to adequately evaluate long-term disease recurrence, progression, and renal outcomes. Therefore, the patient's long-term prognosis remains uncertain, and continued long-term follow-up is warranted. Fourth, the absence of preoperative serum IgG4 testing and renal biopsy limited the completeness of the diagnostic evaluation. Finally, the retrospective collection of clinical data may have introduced information bias. Nevertheless, this case provides valuable clinical insights into the differential diagnosis of renal masses.

## Conclusion

In summary, when IgG4-RKD presents as an isolated renal mass, its clinical and imaging features may closely mimic those of RCC, making an accurate differential diagnosis challenging. This case highlights that, even in the absence of extrarenal involvement, failure to measure serum IgG4 levels preoperatively may result in IgG4-RKD being overlooked and, ultimately, in unnecessary nephrectomy. When suggestive clinical or imaging features are present—such as extrarenal manifestations, bilateral or multifocal renal lesions, lesions centered on the renal pelvis or hilum, impaired renal function, or urinary abnormalities—additional serological testing and percutaneous renal biopsy should be prioritized. Even when there are no extrarenal clues suggestive of IgG4-RD, incorporating serum IgG4 and IgE measurements into the preoperative evaluation and performing a timely needle biopsy in selected patients may help prevent misdiagnosis and avoid unnecessary surgical intervention.

## Data Availability

The original contributions presented in the study are included in the article/Supplementary Material, further inquiries can be directed to the corresponding authors.
